# Industry 4.0 towards Forestry 4.0: Fire Detection Use Case †

**DOI:** 10.3390/s21030694

**Published:** 2021-01-20

**Authors:** Radhya Sahal, Saeed H. Alsamhi, John G. Breslin, Muhammad Intizar Ali

**Affiliations:** 1 Confirm SFI Research Centre for Smart Manufacturing, National University of Ireland Galway, Galway, Ireland; john.breslin@nuigalway.ie; 2 Faculty of Computer Science and Engineering, Hodeidah University, Al Hodeidah 3114, Yemen; 3 SMART 4.0 FELLOW, Software Research Institute, Athlone Institute of Technology, Athlone, Ireland; salsamhi@ait.ie; 4 Faculty of Engineering, IBB University, Ibb 70270, Yemen; 5 School of Electronic Engineering, Dublin City University, Dublin, Ireland; ali.intizar@dcu.ie

**Keywords:** IoT, query, industry 4.0, stream processing, window size, forestry 4.0, internet of forestry things, forest fire detection, forest sustainability

## Abstract

Forestry 4.0 is inspired by the Industry 4.0 concept, which plays a vital role in the next industrial generation revolution. It is ushering in a new era for efficient and sustainable forest management. Environmental sustainability and climate change are related challenges to promote sustainable forest management of natural resources. Internet of Forest Things (IoFT) is an emerging technology that helps manage forest sustainability and protect forest from hazards via distributing smart devices for gathering data stream during monitoring and detecting fire. Stream processing is a well-known research area, and recently, it has gained a further significance due to the emergence of IoFT devices. Distributed stream processing platforms have emerged, e.g., Apache Flink, Storm, and Spark, etc. Querying windowing is the heart of any stream-processing platform which splits infinite data stream into chunks of finite data to execute a query. Dynamic query window-based processing can reduce the reporting time in case of missing and delayed events caused by data drift.In this paper, we present a novel dynamic mechanism to recommend the optimal window size and type based on the dynamic context of IoFT application. In particular, we designed a dynamic window selector for stream queries considering input stream data characteristics, application workload and resource constraints to recommend the optimal stream query window configuration. A research gap on the likelihood of adopting smart IoFT devices in environmental sustainability indicates a lack of empirical studies to pursue forest sustainability, i.e., sustainable forestry applications. So, we focus on forest fire management and detection as a use case of Forestry 4.0, one of the dynamic environmental management challenges, i.e., climate change, to deliver sustainable forestry goals. According to the dynamic window selector’s experimental results, end-to-end latency time for the reported fire alerts has been reduced by dynamical adaptation of window size with IoFT stream rate changes.

## 1. Introduction

Information gathering and transmission are growing to the point where it is common to exchange information between participation in real-time and anywhere in the world. However, the information is required to adjust the process for producing decision-making dynamically. Smart forest is one of the domains that is drastically becoming technologized with data-gathering sites, processing, transportation and analysis. Industrial IoT data-processing poses some unique challenges when endeavouring to make stream processing a reliable solution. Some specific challenges that the IoT industry faces when it comes to data-processing are (1) the retrieval and processing overheads for the huge amount of heterogeneous streaming data generated from a large number of IoT devices, and (2) the missing and delayed events due to unpredictability of data which affects the required rapid response time in the IoT industry [1]. In particular, these IoT devices produce data at a variable rate (e.g., unscheduled events) rather than at a fixed rate, which augments the difficulties for data stream processing platforms to cope with such a sudden increase in streaming rates [2]. Taming this massive streaming data is a very challenging task. Mainly, the true vision of IoT can only be realized if the underlying technologies for stream processing can handle large amounts of data. Realizing the necessity, recently, a few stable and scalable have emerged to facilitate real-time building applications for IoT by processing large IoT data at scale, such as Apache Spark, Apache Flink, Apache Samza and Apache Storm [3] etc. The rapid development of IoT has resulted in the emergence of a few sophisticated query language tailor-made for performing analytics over streaming data, such as Spark SQL, Flink Table API, KSQL, SamzaSQL, and StromSQL. All of these streaming data queries are relying on the concept of windowing to convert continuous infinite data streams into chunks of finite data sets sliced according to a pre-defined time (e.g., minutes, seconds, milliseconds) [4].

Our previous work has addressed the study of the impact of changes in IoT data streams rate over query window configurations [5]. Our evaluation results have indicated a direct impact of any changes in stream rate and window size over the engines’ performance. For instance, consider a fire alarm detection query scenario that processes two input data streams and observes their values at a fixed interval for any fire hazard detection. Input streams/sensors are configured so that they change their stream rate with the changing values (e.g., if the temperature rises beyond a certain threshold, data observation rate is increased to ensure early detection of any fire event). Larger window size facilitates streaming query engines for optimal processing of data with high velocity. Still, it impedes the query engine’s performance due to large intermediate result size or delay events. The smaller window size can lead to short-latency, but it misses events in case of high stream rates. Therefore, a fixed window size will either impede the query engine’s performance due to a large number of intermediate results or miss the detection of fire events due to the small window size. One of the real use case of fire data monitoring is forest fire detection. The forest managers have gradually accepted IoT forest environmental monitoring technologies for taking forest inventory through remote IoT data collection [6]. The IoT-based forest fire monitoring data has the challenge of dynamic ecological changes, i.e., climate change due to unpredictable streaming data. In particular, the timeliness of acquired monitoring IoT data should be considered to avoid real-time data being affected by sudden factors.

**Figure 1:**
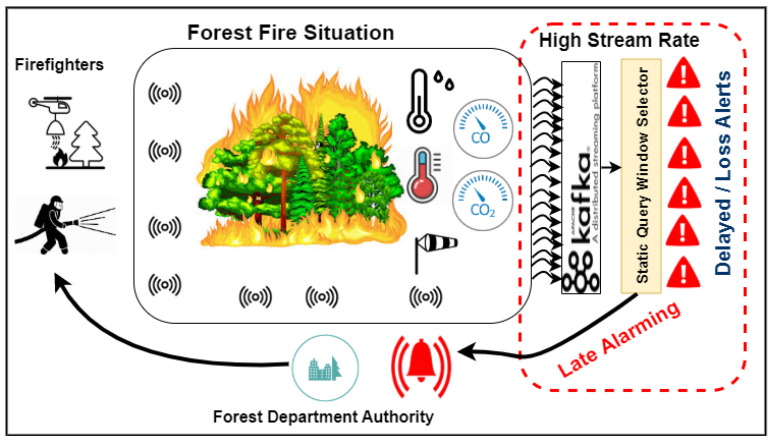
Forest fire monitoring use case using weather sensors data (temperature, humidity, CO and CO_2_).

Internet of Forest Things (IoFT) is version revolution of IoT, which refers to the smart devices distributed in forest for monitoring, management and fire detection and protection. A big picture of the forest fire situation is depicted in Figure 1. As shown, the deployed IoFT is used to monitor weather conditions (temperature, humidity, CO (Carbon Monoxide), and CO_2_ (Carbon Dioxide)). The sensory weather data are collected and consumed by Apache Kafka as a scalable message queuing system. The collected data (i.e., potential fire events) is then sent to the query engine running on top stream processing platform. The number of possible fire events varies drastically during the day, which causes a delayed fire reporting to the forest department authority. The delay will also raise late alarms to the forest department authority to send its firefighters and drones to the burning forest. Therefore, the automation theme of Forestry 4.0 can lead to appropriate, timely action to be taken from the forest department authority side. Applying adaptive window-based streaming data analysis on forest fire data monitoring can provide early warning mechanisms to reduce fire risks by sending quick decision-making responses.

**Figure 2:**
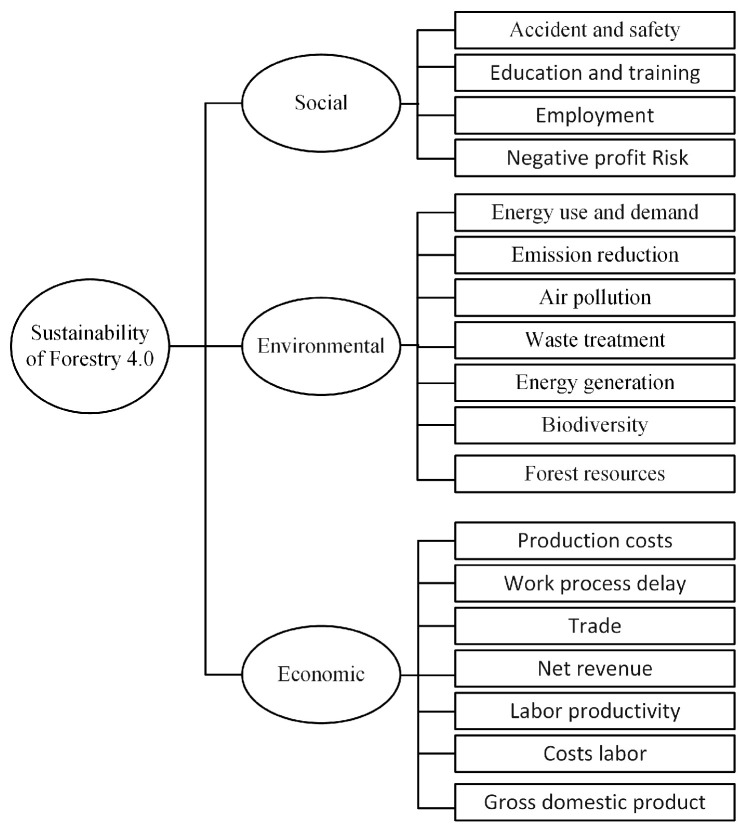
Forestry 4.0 and sustainability impacts.

Sustainable forestry focuses on three impacts, i.e., economic (timber supply), ecological (biological and resilience), and social sustainability (forests multiple-use and non-timber products). The forest operations challenges are finding optimal management strategies to predict the environmental, economic, and social performance of various services, processes and productions. The concept of sustainable forestry is based on sustainable development, which refers to maintaining biodiversity, capacity, productivity, vitality and relevant economic, ecological and social operations impacts [7]. Furthermore, the authors of [8] discussed the challenges of forest operations and the proposed Sustainability Impact Assessment (SIA) framework that analyses the social, economic and environmental. Sustainable forestry focuses on three impacts, i.e., economic (timber supply), ecological(biological and resilience), and social sustainability (forests multiple-use and non-timber products). Figure 2 illustrates the importance of forest sustainability management for economic, social, and environmental. Balancing these impacts is a challenge for improving sustainable forestry in terms of data availability, and quality [9,10]. The authors of [8] summarized the forest operations sustainability impacts in details. Based on the above study, Figure 2 shows the effect and the importance of using techniques solutions for improving Forestry 4.0. Forestry 4.0 aims to bring forest value chain to work within Industry 4.0 parameters (connectivity, security, and productivity, processing remotely). The authors of [11] discussed challenges regarding wireless network availability and connectivity in forest in contrast to industries based on the advanced IoFT, robotics, automation and autonomous technologies.

To the best of our knowledge, there is no dynamic stream processing system that has been studied concerning the forest fire management and detection to manage the unpredictable environmental conditions changes. The dynamic window-based selector is particularly important and relevant for forest fire management and detection use case because of its ability to adapt to the environmental changes in time. It can provide a timely warning about fires by real-time monitoring streaming IoT data to detect the dynamic environmental fire risks. To bring our research work to reality, we develop a data generator to generate IoFT-based forest fire streaming forestry data with fluctuated change rates simulated to rapidly changing in an ecological environment within forestry areas. Therefore, it is sensible to state that our work presents an adaptive stream processing prototype for Forestry 4.0 i.e., IoFT-based forest fire detection can automatically adapt the window size according to several parameters and, thus, improve the performance of the underlying IoFT.

### 1.1. Contribution

The main contributions in this paper can be summarized as follows,

1. We elaborate Industry 4.0 towards Forestry 4.0, which has been proposed as research initiatives in recent years. While most of the publications have focused mainly on the digital technologies, we have focused on applying the automation theme by proposing dynamic stream query processing using IoFT for forest fire detection use case.
2. We provide a flexible model proposing the ideal window type and size depending on stream rates and application requirements. The proposed dynamic query window selector can monitor various external factors including stream rate, resource constraints, and application requirement to propose the most optimal window size and type for a given query. It is also capable of detecting any in-efficiencies and re-deploy the optimal query.
3. We identify and perform a real-world use case of Forestry 4.0 (i.e., forest fire detection based on IoT data) to evaluate the dynamic window selector in the fire situation when the stream rate suddenly changes i.e., weather sensor starts sending streaming data in high rate.

### 1.2. Paper Organization

The remainder of this paper is organized as follows; related work is introduced in Section 2. The  Industry 4.0 towards Forestry 4.0 and the forest fire detection use case is introduced in Section 3 and Section 4 respectively. The research methodology, including the data streaming pipeline and our proposed dynamic window-based selector are introduced in Section 5. The experimental evaluation and discussion are presented in Section 6 and Section 7 respectively. Finally, conclusions and future work are presented in Section 8.

## 2. Related Work

Traditionally, stream processing systems have managed the sudden changes in stream rate by elastically increasing available resources, or by discarding part of the data streams (i.e., load shedding) [12,13,14]. Much work on the potential of stream processing has been carried out in recent years due to the growth in IoT. Our work tackles change of stream rates and window-based stream processing, which are major challenges faced due to loosely couple nature of IoT data streams. The research work of forest environmental management and industry 4.0 including forest fire detection is discussed. Therefore, we divide the existing work into the following categories:

For the stream processing adaptation, Cervino et al. have proposed an adaptive cloud-based approach for provisioning virtual machines with respect to stream rate change [15]. The proposed approach periodically estimates the number of virtual machines required to support the input stream data rate to maintain virtual machines overloaded and meet processing latency. The adaptive stream rate for smart grid applications on clouds has been studied to throttle the rate of generation of power events by smart meters [2].

Furthermore, one of the aspects of the fluctuated streams generated by various IoT devices is the out-of-order events problem. Kun et al. have proposed a real-time query-matching algorithm to generate queries when the number of event types is large and query length is long by minimizing the overhead and reduce the response time [1]. The distributed Platform for Elastic Stream Processing (PESP) has been introduced to deal with changing rates of streaming data [3]. The PESP platform operates a cost-efficient stream processing engines due to a flexible adoption of processing nodes.

With regard to query stream processing, STREAM, a Stanford’s data stream management system, supports a large class of declarative continuous queries over continuous streams and traditional stored data sets [16]. Das et al. have proposed a robust algorithm to automatically adapt the batch size based on the data ingestion rates, variations in available resources, and workload characteristics [17]. Zhang et al. have leveraged adaptive batch sizing and block size to minimize the end-to-end latency of streaming system without prior knowledge of workloads specification [18]. The authors proposed a heuristic algorithm integrated with isotonic regression to automatically learn and adjust batch size and execution parallelism according to workloads and operating conditions.

There has been much work in the area of stream-processing based on querying windows. SABER, a window-based hybrid stream processing system is proposed to adapt scheduling strategy on CPU and GPU with respect to increasing of the share of queries [19]. SPECTRE (SPECulaTive Runtime Environment) is a framework for speculative processing of multiple dependent windows in parallel [20]. The SPECTRE framework has addressed the speculative processing concept to allow the execution of multiple versions of multiple windows using different event sets in parallel. It has provided a probabilistic model to process different window versions that have the highest probability to be correct.

Stream-processing based on sliding window has been extensively studied for different aspects such as aggregations and anomaly detection. For aggregation, the DABA algorithm has been proposed for incremental sliding window aggregation over stream data [21]. Scotty, which is window-based operator has been proposed for aggregation and discretization [22]. The key idea of the Scotty operator is splitting the streams into non-overlapping slices and computes shared partial aggregates per slice while supporting out-of-order processing. For anomaly detection, a sliding window-based strategy has been used for detecting faults over high dimensional streaming data [23]. In particular, the authors has proposed ABSAD approach to select fault-relevant sub-spaces and then detect online faults stream with time-varying characteristics using sliding window.

For IoT forest environmental monitoring, the researchers at Northeast Forestry University have researched a networking based intelligent platform by using the ZIGBEE protocol to monitor their forest environmental factors in time with the new IoT technology [6]. The ZIGBEE-based networking technologies has the advantages of low power dissipation, low data rate, and high-capacity transportation, which makes it more suitable for the design of the node of the forest environmental factors collection platform. In the context of forest fire management, the research works are over-viewed in terms of the satellite systems, optical cameras, and wireless sensor networks detection techniques [24]. The author has discussed several research experiment results and some market product methods for better understanding the fire detection technique stated that each technique has its advantages and disadvantages in terms of efficiency, accuracy, versatility, and other key attributes. Haifeng et al. have proposed a fuzzy prediction algorithm implemented by rechargeable wireless sensor network to assess fire risk and calculated the quantitative potential fire risk [25]. The authors studied the weather variables including temperature and humidity as input of the proposed fuzzy prediction using 24-h monitoring of whether meteorological factors. They concluded that it is difficult to predict the occurrence of forest fires accurately.

In the field of IoT-based applications and fire management, Faisal et al. have designed a wireless sensor network using multiple sensors for early detection of house fires [26]. The authors have used the Global System for Mobile Communications (GSM) to avoid false alarms and they have tested their system by simulating a fire in a smart home using Fire Dynamics Simulator. For the IoT-based forest fire detection, the proposed work in [27] provides new improvements such as the use of innovative IoT technologies and a data treatment focused on the prevention, detection, activation of alarms and management of operations for the extinction of fires. The authors have developed a system with secure communication which has been configured for monitoring different variables of environments including temperature, humidity, CO, CO_2_ and wind speed. Furthermore, the authors of [28] addressed the techniques used for reducing pollution such as CO_2_ for improving smartness application in the real world via collaboration of drone and IoT framework applications. However, the authors of [29] presented the collaboration of smart IoT devices and drone for improving emergency response.

However, some good research work has been carried out to the dynamic stream processing and previous work has not comprehensively considered dynamic stream rate and query windowing using an open knowledge base to recommend the proper window configuration. According to the authors’ knowledge, no comprehensive work towards Forestry 4.0 which exploits the capabilities of streaming data platforms to manage the forest fire due to dynamic rapidly changing of the ecological environment. So, we proposed an adaptive window selector to dynamically change window configuration to face stream fluctuating. Then, we defined a real-world IoT-based use case, fire forest detection, as a dimension of Forestry 4.0 using the power of streaming technologies to provide a timely warning about fires by real-time monitoring streaming IoT data to detect the dynamic environmental fire risk.

The wood resource is from the forest, making forestry important for economic, cultural, and ecological. Based on industry 4.0, the authors of [30] introduced the concept of forestry 4.0. they showed the implementation of forestry 4.0 with multi-domain systems. The authors of [31] discussed the advantages and limitations of using IoT for wood processing in industry. Many studies discussed industry 4.0 in several field including economic and business [32], information sharing [33], technologies and applications [34], future trends [35] and industry 4.0 in the wood industry [36] (https://awfsfair.org/2019/04/industry-4-0-in-the-wood-industry-beyond-the-buzz/). However, many studies have been done in connecting industry 4.0 and forestry 4.0 with the help of advanced technologies such as IoT, Artificial intelligence, robots, vehicles and etc. [37], industry 4.0 application to forestry [30], wood processing [31], digitization in wood supply [38]. The authors of [39] discussed Internet of improving the forest sustainability. Moreover, the authors of [40] introduced the framework of industry 4.0 from the prospective of forest supply chain. The framework components were included system intelligence, digital technologies, communication network infrastructure, collaborative supply chain of forest.

Forest fire accident represents a common hazard that destroys the forest. Therefore, giant trees were reduced drastically, which led to an unhealthy environment for human beings and animals. The authors of [41] introduced IoT devices and cloud to produce forest fire alert in case the fire is detected. Therefore, detecting fire is necessary to avoid fire hazards in the forest and benefit from distributing IoT devices in forest areas. The authors of [37] introduced optical remote sensing for early fire alert systems. The proposed system architecture included spaceborne, airborne, and terrestrial to detect fire with a high accuracy level. Smart IoT devices are implemented in smart systems to measure CO_2_ emissions of different forest fire sources [42]. Furthermore, IoT devices are used for designing efficient forest fires detection [43]. The summary of the related research work has been presented in Table 1.

## 3. Industry 4.0 towards Forestry 4.0

Industry 4.0 is considered the fourth industrial revolution introducing a new paradigm of digital, autonomous, decentralized control for manufacturing systems. The concept of Industry 4.0 refers to smart manufacturing toward to digitization, collaboration and automation. The authors of [48] identified the component of industry 4.0 include Cyber–Physical Systems (CPS), smart factory, IoT, and internet of services (IoS). CPS Refers to the fusion of physical world in Industry 4.0, while IoT refers to the connectivity between the physical elements in industry 4.0. Smart factory refers to all categories of smart phyiscial components such as devices, robots, computers, cameras sensors, and etc. IoS refers to the processing and functions of all smart devices connected via IoT. Moreover, the Industry 4.0 impacts can be improve socially, economically, and environmentally [48]. Furthermore, Industry 4.0 covers a broad range of technologies, processes, and systems mainly related to industry digitalization. In terms of data-related technologies, the main areas of Industry 4.0 are CPS, Industrial Internet of Things (IIoT), Cloud Solutions and Decentralized Services, and  Big Data and Stream Processing technologies for processing large amounts of production data in real-time [49].

**Figure 3:**
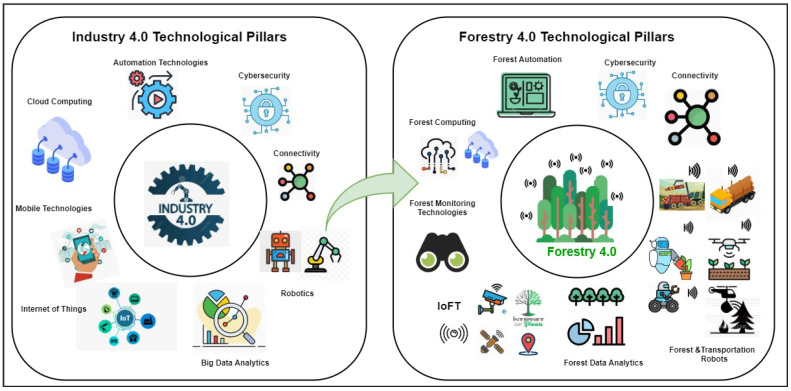
Transformation of Industry 4.0 technologies toward Forestry 4.0.

The transfer of Industry 4.0 concepts and technologies to the forestry sector appears to be a promising way to optimize existing processes and to spawn new business models. Forestry 4.0 concept is inspired by Industry 4.0 concept, which plays a vital role in the next industrial generation revolution. Internet of Forest Things, AI, automation, smart devices, Blockchain and digital twins will drastically change the Forestry 4.0 for the better. These advanced and emerging technologies are used to solve the operational issues related to create a sustainable Forestry 4.0 (https://www.woodbusiness.ca/final-cut-forestry-of-the-future-the-sustainable-revolution). Furthermore, the combination of emerging technologies for sustainability is the efficient way of Forestry 4.0 future toward Industry 4.0. For Forestry 4.0, network performance and communication network are required such as IoFT, wireless sensor network, IoT, big data, edge computing, drone, and cloud computing. Smart IoT device, mobile devices, IoFT, smart devices, robots, objects, vehicles, drone, and machines; as shown in Figure 3. The authors of [40] have defined the Forestry 4.0 as paradigm of forest industry (digitization, connectivity, harvesting, automation and transportation). It focused on digitization of end-to-end smart devices as well as customers. Thus, the forestry 4.0 concept combines digital technologies, network connectivity, processing and operations, and collaboration.

Furthermore, Forestry 4.0 technical realization involves connecting wood resources, data sets, existing and new hard and software components, and stakeholders into a novel IoT, Services, and People in forestry [30]. Based on the manufacturing industries experience with Industry 4.0, Forestry 4.0 concept has been launched by FPInnovations (https://web.fpinnovations.ca/) as an initiative for digitalization in the Forest Industry. Forestry 4.0 initiative aims at enabling the upstream part of the forest value chain in Canada to fully leverage the agility and power of the fourth industrial revolutions. The development of Forestry 4.0 achieves solutions for issues that affect the forest industry including labour shortages, performance, forest connectivity, safety, environmental performance improving, sustainability and reducing costs. IoFT is based on big data gathering and exchange, real-time connection, and assembly of technologies. Implementation of communication among the distributed smart devices in the forest environment is the aim of the Internet of Forest Things, enabling the industry 4.0 implementation standard. Therefore, communication for wide range requires device-to-device, robot-to-robot, vehicle to vehicle, vehicle and robot to infrastructure things, human to devices and machine, interconnected things among heterogeneous devices, and cellular to operations via Internet network [50,51]. The focus of the IoFT is on keeping connectivity links among all forest components (robots, smart devices, machines, vehicles, devices, operations, cellulars, etc.) in large forest areas. The authors of [39] discussed the smart devices and connectivity for digital Forestry 4.0 and monitoring applications considering sustainability. The Internet of Forest Things is the key enabling to exchange real-time information between Forestry 4.0 operations components and decision centre and industry 4.0. Furthermore, applying the IoFT can monitor forest environment impacts in real-time with intelligent platforms. The authors of [50] showed many benefits of using the IoFT to improve Industry 4.0 via low data rate, transportation of high capacity, low power consumption and efficient gathering data. Moreover, Internet of trees is used to monitor and early fires detection (https://https://electronics360.globalspec.com/article/11399/internet-of-trees-early-detection-of-forest-fires/), and fight climate change (https://www.euronews.com/living/2020/07/29/internet-of-things-technology-is-being-used-to-help-trees-fight-climate-change).

Forestry 4.0 concept, four research themes have been defined which, through their distinct functions; the real environment, IoFT, the next-generation fibre supply chain, data analytics [11]; as shown in Figure 3. For the real environment, the forest supply chains, accurate information is needed on the amount and quality of fibre available, the physical environment in which operations will need to be deployed and the transformational outcomes of the various phases of harvesting systems. In regards to forestry data-related technologies, data are collected through remote sensing, satellites, drones or aircraft, imagery and LiDAR 3D cloud points, infrared cameras, high-resolution camera, etc. For the Internet of Forest, the forestry industries face the most significant challenge regarding communicating in remote areas with a high cost of satellite communication. Therefore, the Internet of Forest which refers to various machines’ connectivity, is used as a collaborative system based on real-time communication between machinery, infrastructures and digital devices to control operations, even remotely. For the next-generation fibre supply chain theme, the advanced technologies will be required in harvesting systems to truly enable full Forestry 4.0 functionalities around connectivity, automation, and agility to upstream and downstream changes in the supply chain. For automation, the production chain must be updated using the latest technological developments, such as sensors, augmented reality devices, more autonomous intelligent transportation systems (self-driving vehicles). For data analytics, forest management’s decision-making process must take account of analyzing vast data (such as geographical or geological data or those referring to wildlife biology). The forestry data analyzing is beneficial to early inform and warn for risk analyses, accident statistics, timber products supply chain, forest-damage, forest fire, etc. Forest fire is one of the risks which has significant damage to the environment which motivates us to identify and perform it as a real-world use case (i.e., forest fire detection based on IoFT data) as a dimension of Forestry 4.0 using the power of streaming technologies. In particular, IoFT streaming forestry data analysis can support and automate early warning systems that ensure protection against forest fires around the clock have replaced forest workers and volunteers doing duty on watchtowers. The summarized comparison of industrial research in forest fire detection domain has been described in Table 2.

**Figure 4:**
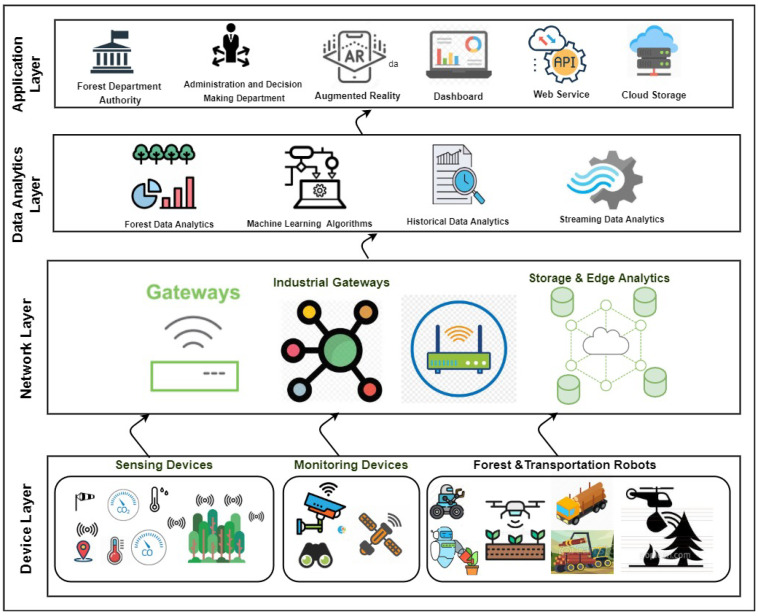
Forestry 4.0 layers.

Based on the above, we try to build Forestry 4.0 in several layers based on [52]. Figure 4 illustrates the forestry layers, including smart devices layer, network layer, data analysis layer and application layer. Each layer contains various devices, technology, and technique to build smart Forestry 4.0 to be automated, digitalization and collaboration. In the forest layer, forest world devices are used for sensing, monitoring, forest robots and transportation robots. Network layer refers to advanced and emerged communication technology such as 5G and 6G technology that can make the interaction between devices reliable and without human intervention. Gathered data are processed in the data analyses layer. People and employers can monitor Forestry 4.0 in the application layer.

## 4. Forest Fire Detection Use Case

As a use case, IoFT aims to use different smart devices to measure forest parameters CO, CO_2_, monitoring, temperature, detecting fire, RFID, sensors, cameras, etc., without human intervention; as shown in Figure 4. These devices send the gathered data into the centre platform via advanced wireless communication technologies. Industry 4.0 people or employers in the centre platform can interact with smart IoFT devices, process received data, estimate forest growth, monitor trees’ health, and fire detection. Implementing Industry 4.0 technologies reduces data collection cost, improves sustainability, monitors forest utilization resources, and measures forest parameters. Therefore, decision-making can be real-time and easy, while growth forest prediction can be more accurate and reliable due to continuous measurement. These will play a vital role in improving economical impact, environmental impact of Forestry 4.0.

**Figure 5:**
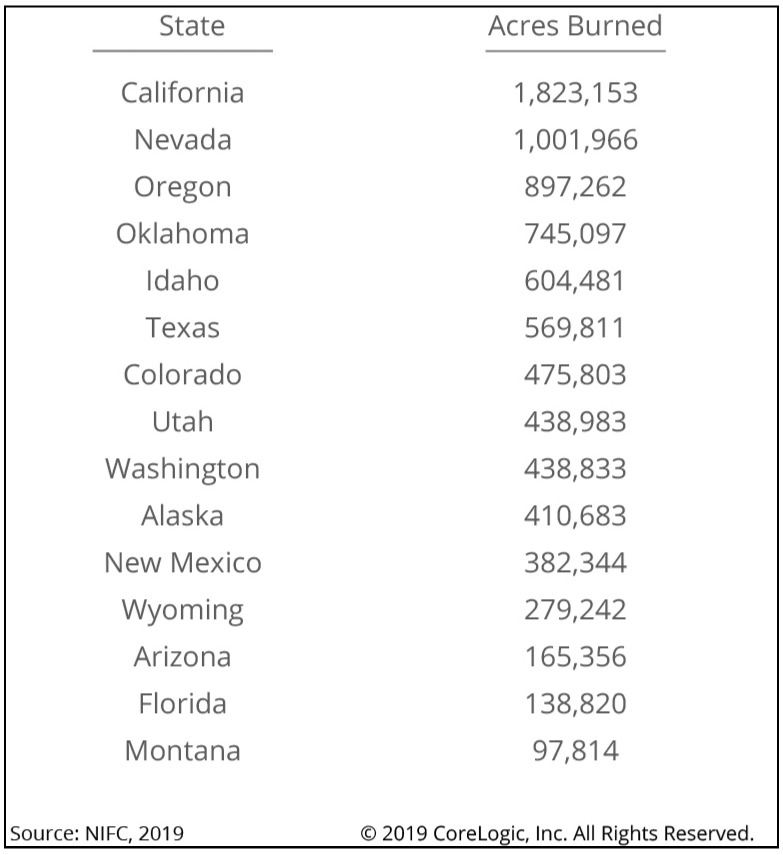
Top 15 states based on wildfire acreage burned in 2018; NIFC 2019.

Forest fires, which are also called wildfires, are among the greatest disasters in the world today. In 2018 alone, 8,767,492 acres burned, roughly equivalent to 74 of the 75 largest cities in the United States combined. It is the sixth-highest total since modern historical records began in the mid-1900s, indicating that no state is entirely free from wildfire risk in the US. CoreLogic Wildfire Risk Report for 2019 highlights that the total estimated reconstruction cost value for the extreme-risk homes is more than $221 billion, with California metropolitan areas dominating the top 15 risk regions (see Figure 5). In late August 2019, for example, Brazil’s National Institute for Space Research said that the number of fires in the country (i.e., Amazon) largely set by humans had jumped 84% in 2019 over the same period in 2018 (https://fortune.com/2019/08/25/causes-of-amazon-forest-fires/).

**Figure 6:**
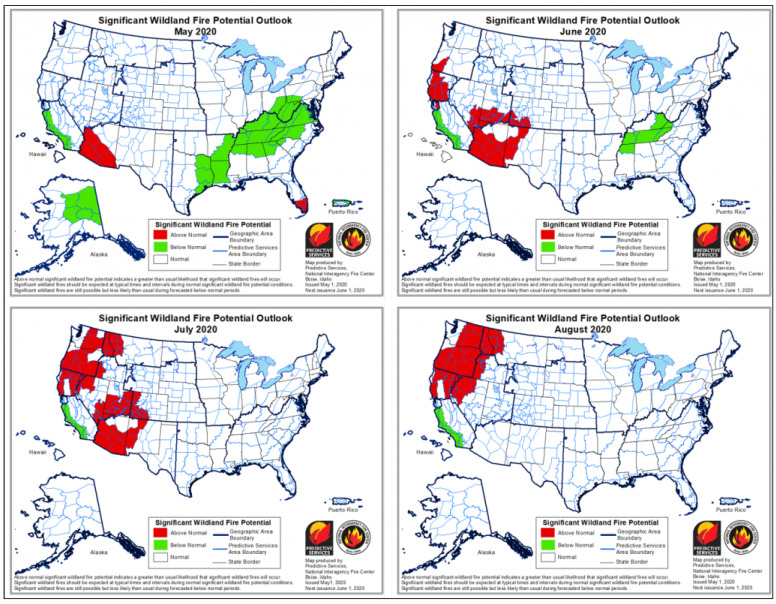
Forest fire outlooks in 2020 for May, June, July, and August, highlighting the elevated risk in the Pacific Northwest, Northern California, and the Southwest throughout the summer.

The year 2020 has been a year like no other due to COVID-19, which will change the world forever. As the scientists, researchers, the World Health Organization, and social communities fight the pandemic, another crisis is unfolding worldwide. Figure 6, (https://cleantechnica.com/2020/05/13/2020-fire-season-covid-19-not-a-match-made-in-heaven/) depicts the forest fire outlooks in 2020 for May, June, July, and August, highlighting the elevated risk in the Pacific Northwest, Northern California, and the Southwest throughout the summer.

Substantially, a forest fire happens due to rapidly changing ecological environments such as uncontrolled climate changes, making the forests unable to recover from devastating consequences for the long-term. For example, the climate changes cause to change the soil moisture and surface temperatures, making the soil becomes water repellent [55]. The forest department authorities’ enormous challenge is that the forests are usually remote, abandoned/unmanaged areas filled with trees and affected by dynamic environmental variables, e.g., temperature, humidity, CO, and CO_2_.

This issue has been a research interest for many years; many very well-studied solutions are available out there to propose an effective way to minimize the damages caused by the fires. Early detection of forest fires is the most attractive trend for the market and research, making decision-makers take a fast appropriate reaction. There are several forest fire detection techniques, and monitoring systems employed by authorities, including human-based observation, satellite-based monitoring systems, optical camera-based monitoring systems and wireless sensor networks [24]. The human observation is inefficient due to the error-prone. It provides an accurate forest fire prediction. As humans get fatigued by time, their forest fire prediction will be inaccurate due to less considering environmental impact, making it a non-reliable solution to reduce forest fire risks. The promoted satellite monitoring systems suffer from severe limitations failing speedy and effective control for forest areas. For example, the satellite systems may not be available for continuous-time to cover the full regions within forests such as gaps in time when the satellite is not within the field of view from certain regions or spots of the forest. On the other hand, the optical camera-based monitoring systems are costly in building towers and communication infrastructure in the forests’ remote areas. Furthermore, the optical camera-based monitoring systems may provide false alarms due to night vision and weather conditions such as wind-tossed trees and cloud shadows that affect camera performance.

Recently, wireless sensor networks are considered the best available solution for forest fire detection. They can provide all the required information that influences the environment at any moment accurately. The wireless sensor networks are easily connected and deployed in broad and inaccessible forestry areas. Accordingly, the researchers and industries have shifted to IoT paradigm, which has been conducted in various fields. For instance, the proposed work in [27] provides new improvements such as the use of innovative IoT technologies and a data treatment focused on the prevention, detection, activation of alarms and management of operations for the extinction of fires. The authors have developed a system with secure communication configured for monitoring different variables of environments including temperature, humidity, CO, CO_2_ and wind speed. Lately, the forestry companies leverage the agility and power of the fourth industrial revolution (i.e., Industry 4.0) towards Forestry 4.0 to utilize the IoT sensors capabilities which send real-time streaming information to early detect wildfire.

Forest fires can happen due to climate change, and cause significant environmental damages. So, we identified a forest fire detection use case as one of the dynamic environmental management challenges. Several detection and monitoring systems are used by authorities to detect the fire as fast as possible, and its exact localization and early notification. As many IoT devices are working together to detect forest fire, the fire alarm detection technologies can help support the decision-making process due to a rapidly changing ecological environment. In particular, the generation of IoT streaming data technology allows managers to establish a set of early warning mechanisms for the quick response and decision making, together with having full use of the data on environmental performance evaluation.

## 5. Methodology

**Figure 7:**
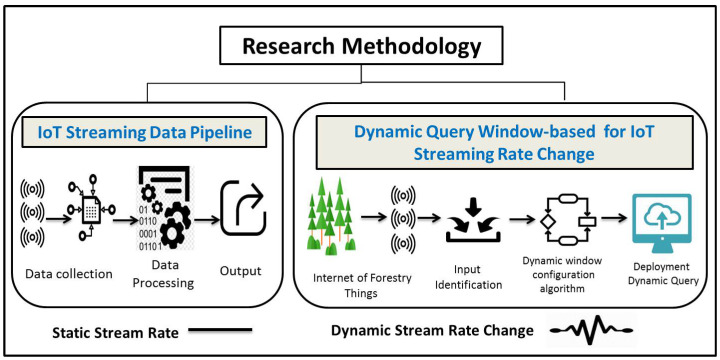
Research methodology description.

**Figure 8:**
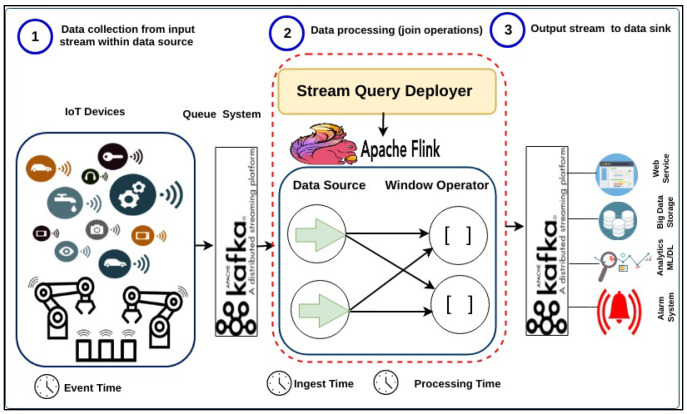
The workflow of data stream pipeline over Apache Flink consisting of three phases (1) data collection (2) data processing and (3) data output.

This section will describe our approach to investigate the dynamic stream query processing using IoT-based forest fire detection use case. To do so, we will introduce The importance of dynamic window sizing over IoT-based stream rate change and then two data streaming pipelines, which are static stream rate and dynamic stream rate change (see Figure 7). The data stream processing pipeline for static stream rate includes IoT streaming data pipeline over Apache Flink (data collection, data processing, data output) and its evaluation, described in Section 5.2 and Figure 8. Then, the dynamic query window-based selector for stream rate change includes the problem of the dynamic streaming query and the proposed dynamic window-based selector for streaming query (dynamic window configuration algorithm), described in Section 5.3 and Figure 12.

### 5.1. The Importance of Dynamic Window Sizing over IoT-Based Stream Rate Change

As IoT streaming data is potentially never-ending, analyzing such data can be never-ending as well. Therefore, the stream windowing concept is re-emerging and being used in IoT-based data stream processing [4]. All stream queries are executed as multiple executions of the same query over data within a single window. Hence, the repeated execution of queries on changing data within the window is the major resource-intensive task. Performing analytics (i.e., aggregation) over streaming data makes it further resource-complicated, bringing the windowing function into further limelight. Hence, any stream processing system’s performance depends on the configuration of windows, including their type and size. A larger window size is used to facilitate streaming query engines for optimal processing of data with high velocity to deal with higher stream rate. However, the larger window size increases the latency between the input arrival time and output generation in lower stream rate. Furthermore, a few external factors can also impede a query engine’s performance, such as application requirements, resource configurations, number and type of queries, etc. Therefore, it is of utmost necessity to maintain an optimal windows size at the query deployment time and during the executions of queries over the time. As the query static window configuration may cause unreasonable high latency in case of low stream rate or a resource contention problem and/or violate workload requirements in case of high stream rate.

However, due to multiple external and dynamic factors it is not possible to guarantee an optimal window size and type which could be valid throughout the life-cycle of a streaming query. Ideally, the stream query processor must cater to external factors such as stream rate and resource availability to continuously monitor the performance and recommend the alternative optimal solution as soon as it is available. Consequently, if the stream processing platform is windowing that data for processing, the windows no longer represent the data that actually tremendously arrived. In particular, alignment of stream rates with window-based analytics is difficult, especially in cases where these analytics are required for sending preventable emergency alerts to avoid downtime and costly unplanned maintenance.

Indeed, not only the windowing configuration affects the stream processing system performance, but also fluctuations of the incoming streaming data rate [20]. Consequently, in this work, we will study the data drift in IoT typically faced by industrial data analytics, and discuss Apache Flink stream processing windows. Flink stream query processing is insufficient to execute the same continuous query on variable stream data (i.e., high-rate and low-rate). Significantly, choosing the proper window size in terms of milliseconds, seconds, or minutes according to stream rates is beneficial to avoid data drift problems to meet analytics requirements such as latency constraints. We study different stream rate scenarios with regards to window size such as (1) assigning small window sizes for high stream rates to materialize enough tuples (sequences of values) to be joined and (2) assigning large window sizes for low stream rates that could buffer enough tuples for insightful joins. Then, we provide a flexible model to propose the ideal window type and size depending on stream rates and application requirements.

In this paper, we performed an empirical study to showcase how constant window size and type impede query engine performance whenever stream rate varies. We evaluated the impact over the Flink query engine’s performance with changing stream rate while keeping the window size and type constant. Advocating the need of adaptive stream query engine, we proposed a dynamic query window size and type selector, which can monitor various external factors including stream rate, resource constraints and application requirement to propose the most optimal window size and type for a given query. The proposed solution is also capable of detecting any in-efficiencies and re-deploy the optimal query. A real-world use case of forest fire monitoring based on IoT data is selected to evaluate the dynamic window selector in the fire situation when the stream rate is changed i.e., weather sensor start sending data in high stream rate.

### 5.2. Data Stream Processing Pipeline

This section presents a general overview of the data stream processing pipeline for a query engine used in the Flink streaming platform. Then, we discuss the different types of windows.

#### 5.2.1. IoT Streaming Data Pipeline over Apache Flink

A typical data pipeline scenario could be as collecting generated data from IoT devices by Kafka once the events are observed. Then, the Kafka produced data are ingested into Flink by Flink consumers. The Flink executes the deployed continuous join query over the ingested data by allowing the running windows to trigger its output based on its timestamps. The processed output stream will then be sent again to Kafka to be consumed within the data application pipeline. In doing so, the workflow for IoT data stream pipeline, shown in Figure 8, consisting of three phases which are; (1) data collection (2) data processing using Apache Flink) stream data output. Each stage can be described as follows:

In the data collection phase, the input data stream is gathered from IoT devices (i.e., data source generators) using Apache Kafka as a scalable message queuing system. Apache Kafka is responsible for sending input streams to Flink and receiving output stream from Flink. Worthwhile, other distributed queuing management technologies such as RabbitMQ, Amazon Kinesis, Microsoft Event Hubs, and Google Pub/Sub. We chose Kafka as it is the state-of-the-art, distributed large-scale real-time data applications. It has high delivered and ordering guarantees of the data stream, making Kafka reliable in data drift situations.

For data processing over Apache Flink, this phase contains two components; stream processing engine used Apache Flink (https://flink.apache.org/), and stream query deployer to deploy a stream query, i.e., join stream query over Apache Flink. The choice of using Apache Flink to implement this phase can be attributed to three characteristics of its approach. First, it integrates with the best existing ideas in the industry, such as the open-source adopter of the Dataflow/Beam model and compatible with Kafka to guarantee the reliability of the data pipeline with different stream rates. Secondly, it has a powerful consistency via snapshots, savepoints, and streaming SQL. Third, it is a highly-scalable for the world’s most demanding stream processing applications. It can scale to thousands of cores and terabytes of application state and deliver high throughput and low latency for latency-critical applications. Fourthly, it is continuously improving for streaming processing across the industry around the globe such as Alibaba, eBay, Lyft, Uber... etc. Fifth, it is automatic scaling with the fluctuated data stream rate.

In the output stream to data sink phase, the output stream is sent back again to Kafka to be consumed by other data sinks. These data sinks could be any data consumer within the real-world application in the industrial setting such as web service, real-time dashboard, storing in big data storage (e.g., HDFS, MongoDB), and sending to alarm systems. Moreover, the output stream could be passed to the next data analytic phase, such as machine learning and/or deep learning.

#### 5.2.2. Query Window-Based Streaming Data

The join query is a common and well-understood operation in batch and stream data processing to connect the rows of two relations and/or streams. Fink provides two main join types; regular and time-windowed. Regarding this work, a time-windowed join is considered which joins two input streams within specific time constraints, i.e., a time window. The join window operation matches the elements of two streams that share a common key within the same window. According to the time-streaming context, Fink provides three types of windows to join operations on finite size of time-stamped data including, tumbling, sliding and session. A tumbling window is a series of fixed-sized, non-overlapping and contiguous time intervals. Sliding window assigns elements to windows of fixed length; hence, sliding windows can be overlapping if the slide is smaller than the window size. Session window groups events with session activity gap.

**Figure 9:**
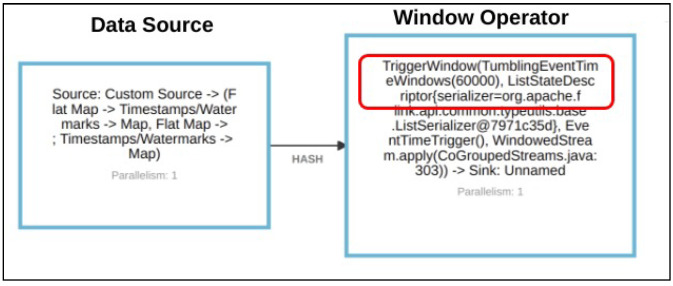
Example of deployed join stream query on Apache Flink web dashboard including data source and window operator using tumbling window for 1 min, i.e., 60,000 milliseconds.

Without loss of generality, Figure 9 describes the translated Flink job of join query which contains data source and window operator. The Flink tumbling window operator splits the stream into buckets of finite size based on the configured window size, 60,000 s and then triggers the output when the time passes the pre-defined watermark.

#### 5.2.3. Evaluating the Impact of Changes in IoT Data Streams Rate over Query Window Configurations

In this section, we overviewed the evaluation of the impact of any changes in stream rate and window configurations over the performance of a stream processing engine which was addressed in our previous work [5]. In particular, we implemented an IoT data stream pipeline over Flink to consume data from Kafka, ingest it into Flink, execute inner joins over Flink query engine, and then send back the joined streaming result to Kafka be consumed by other data sinks.

We conducted our experiments using two input data streams which are streamed using Kafka and processed by Flink. Query results are stored in MongoDB as data sinks. We evaluated three types of windows supported by Flink, namely tumbling, sliding, and session. For the first input stream, we varied it to 10 different stream rates i.e., 60, 120, 180, 240, 300, 360, 420, 480, 540, and 600 tuples per minute. For the second input stream, we only produced one tuple per minute at a fixed rate. We assess the latency (time consumed between the input arrival and output generation) using two window sizes; (1) the small window size which is one minute denoted by 1-min, (2) the large window size is five minutes denoted by 5-min. To consider the queue delay for Kafka, we calculated the latency of window-based join queries executed on Flink as (1) Flink with Kafka (i.e., the time consumed between the event observed until the output is generated), denoted by Flink w/Kafka and (2) Flink without Kafka (i.e., the time consumed between the event is already ingested into Flink till the output is generated), denoted by Flink wo/Kafka.

**Figure 10:**
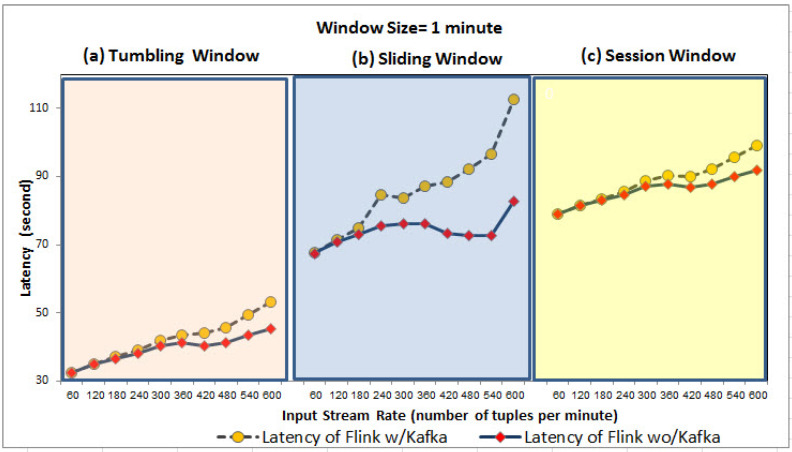
The latency of Flink join using window size 1-min.

**Figure 11:**
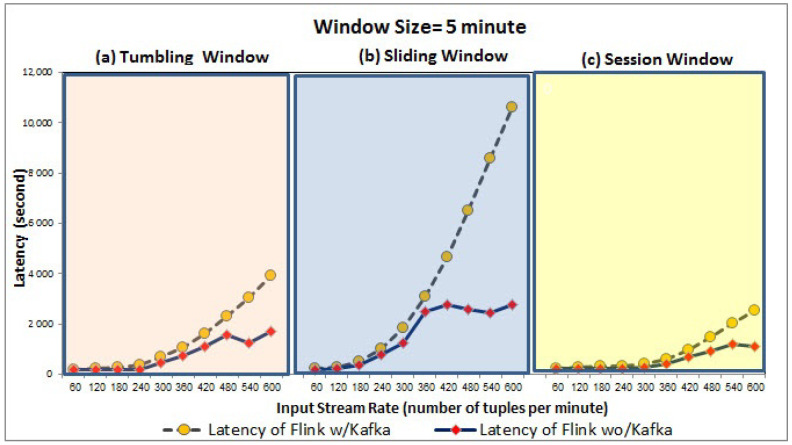
The latency of Flink join using window size 5-min.

As shown in Figure 10 and Figure 11, Flink w/Kafka and Flink wo/Kafka latency of queries increases linearly with the increase in stream rate for both 1-min and 5-min window sizes for three window types. In particular, the Flink w/Kafka grows fast due to Kafka’s delay in launching Flink Kafka consumers to consume the input streams and Flink Kafka producers to send back the output stream to Kafka. However, the average latency of Flink w/Kafka over 5-min window is longer than the 1-min because the ingested tuples (i.e., from the first minute to the fifth minute) wait until the Flink window triggers its output. The larger window size can result in large data size ingested into Flink, and when this large data is processed, it incurs higher processing time/latency. Our evaluation confirms the impact of dynamic stream rate or other factors over the query engine’s performance. However, there is no one window size and/or type fitting all input stream rates. Our evaluation strengthens the case for adaptive window type and size recommendation based on the input stream rate variation.

### 5.3. Dynamic Query Window-Based Selector for Stream Rate Change

In this section, we first describe the formulation of the investigated problem of stream query window-based configuration regarding stream rate change. Taking advantage of the learned lessons from the conducted experiments in our previous work (see Section 5.2.3 [5]), a prototype of a dynamic window-based selector for stream query running over fluctuated stream rate is also presented.

#### 5.3.1. Problem Formulation

Many factors affect stream query processing performance, such as varied stream rates, workload requirements, and resource limitations. According to our windows-based stream analysis, the window configuration in the streaming query (i.e., type and size) significantly affects the performance of the query engine. Assuming that the stream structure remains fixed and the query and the required resources are available according to the data application needs.

A proper window configuration is required for stream query running on various stream rates by estimating factors related to the stream analytic process. These factors could be called input parameters for querying window configuration over different stream rates. Some parameters could be identified priory using collected statistics from the past analytic process (i.e., queries) such as schema, stream rate behavior and previously executed job statistics. Some of the parameters will be identified by users such as resource capacity and application requirements, i.e., latency, accuracy, data enrichment, etc. The other parameters values could be captured during running stream queries on a stream processing system such as live stream rate and current job status. These parameters could be described in vectors as follows.

$$Stat_{histo}=\left[streamrate,streamjobstatus\right]$$

$$Stat_{live}=\left[streamrate,streamjobstatu\right]$$

$$Req=[rq_{1},rq_{2},...rq_{i},...,rq_{n}],1<=i<m$$

$$Res=[rs_{1},rs_{2},...rs_{i},...,rs_{n}],1<=i<n$$

where *Stat*${}_{histo}$ and *Stat*${}_{live}$ vectors denote the collected historical stream statistics from the past streaming jobs behaviour and the live streaming statistics including stream rate itself and current streaming job status respectively. *Req* denotes the application requirements for the desired analysis, such as the maximum number of produced tuples known as data enrichment. Data enrichment is a process of data collection to provide a richer profile that will be used for further data analysis, such as machine learning and deep learning models that require large sets of data. *Res* denotes the number of given resources to the stream processing system including CPU, memory and network bandwidth etc.

#### 5.3.2. The Proposed Dynamic Window-Based Selector

**Figure 12:**
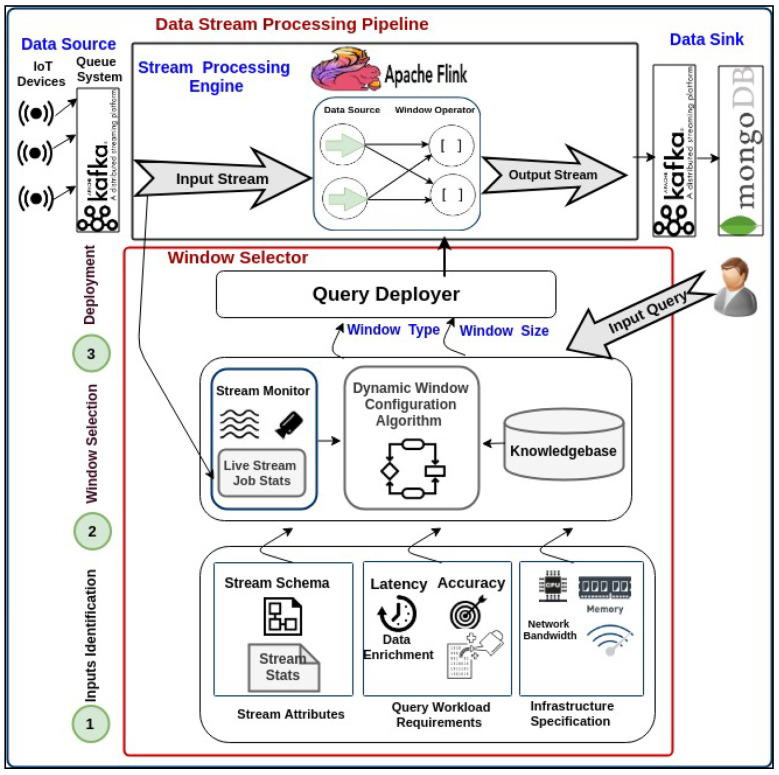
The prototype of a dynamic window-based selector for stream query running over fluctuated stream rate consisting of three phases: (1) input identification; (2) window selection processing and (3) query deployment.

The dynamic window-based selector is proposed to dynamically select the proper window configuration for a stream query based on the change of stream rates concerning the relation of stream attributes, workload requirements, and infrastructure specification. A typical scenario of running stream query could be deploying the input query with an initial window configuration. The stream query engine is initially configured the querying window concerning the identified inputs including stream attributes, workload requirements, and resources capacity. Then, the behaviour of stream rates is monitored, which leads to a new deployment of window-based query configuration. The proposed prototype contains three phases, including (1) input identification, (2) window selection and (3) query deployment. Each phase can be described as follows (see Figure 12).

**Inputs identification phase.**

The relevant inputs are identified in this phase, including the stream attributes, the query workload requirements, and the infrastructure specifications.

The stream attributes represent the stream’s schema (i.e., stream structure) and the historical stream statistics (i.e., historical data collected during the stream ingestion in the past). The stream structure could be drifted when the data schema changes at the source, such as adding, deleting, and modifying the type of fields. We opted the stream structure as a fixed schema to keep the problem of stream rate change tractable regarding our work.

Three workload requirements are considered; latency, accuracy and data enrichment. The latency maintains when an event is observed and when the results corresponding to that event are generated. So, the latency depends on window size, which means that the small window size leads to low latency materialized output. In contrast, the large window size has long latency due to late output materialization. The data accuracy refers to the data quality (i.e., data values are correctly stored). According to this work’s scope, the inconsistencies of data can be caused by the stream rate change. Due to the granularity of the stream rate, the data could arrive out-of-order, affecting the data processing and the accuracy of the output. Consequently, a correct stream query window configuration can adapt with stream change to keep the output stream accurate over various stream rates. Intuitively, the generated output stream rate increases monotonically with the increase in the input stream rates. Thus, the output stream rate should be enriched in case of a high input stream rate. As reported by the study in subSection 5.2.3 [5], not all query window configurations over high stream rates lead to enriching resulted stream. Therefore, picking the proper query window configuration to meet the data enrichment requirement is very important, and it should be considered whenever a stream query is deployed.

Declaring the infrastructure specifications (i.e., CPU, memory and network bandwidth) is essential for decision-making processes in query stream processing. A prior knowledge of the available resources can help the query stream engine select the appropriate query configurations and avoid resource limitation problems in the case of unpredictable stream rates.

**Window selection phase.**

The window selection phase contains three components i.e., stream monitor, knowledgebase and dynamic window configuration algorithm.

Stream monitor includes monitoring systems to observe different input stream characteristics, including its rate, schema and other relevant information. The stream monitor builds statistics on the go for live stream jobs being executed by the query engine. Both the input stream rate and the current status of live jobs help the optimizer to choose the suitable window configuration whenever the stream rate fluctuates.

**Figure 13:**
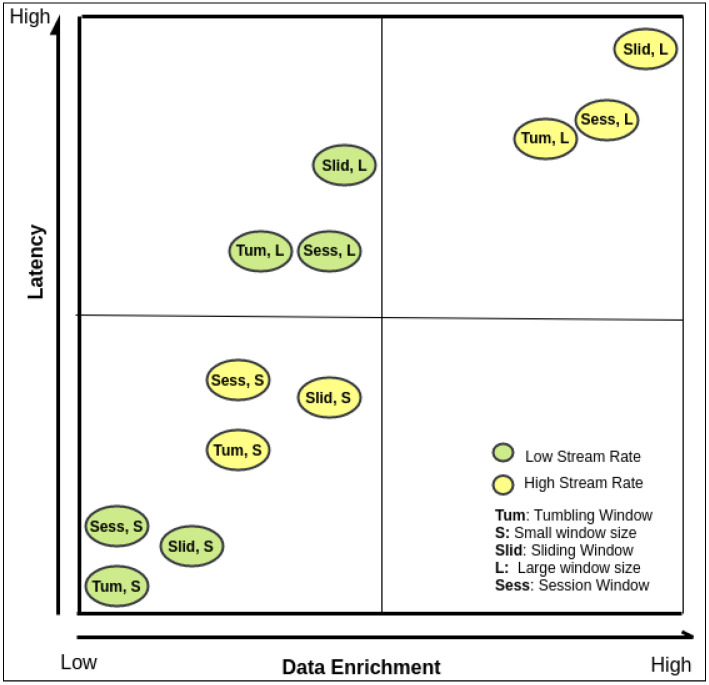
The four-quadrant matrix model of stream query window-based configuration, i.e., type and size concerning the workload requirements including data enrichment and latency.

The knowledgebase contains the previous recommended window configurations of different stream rates, including window type and size regarding the relationship between the identified inputs, query workload requirements and infrastructure specifications. We built the knowledgebase to get recommendations for window configuration regarding workload requirements, including latency and data enrichment over stream rate changes. More specifically, Figure 13 depicts the four-quadrant matrix model of window configurations, including type and size for low and high stream rates. The quadrant matrix model represents the information extracted from the conducted experiments in the study in Section 5.2.3. Two workload requirements are carried out on different stream rates which are latency and data enrichment. To simplify the evaluated results shown on quadrant matrix model (i.e., decision making tool), we scale the 10 generated input stream rates into the following two categories (see Section 5.2.3); low stream rate which comprises of first five stream rates i.e., 60, 120, 180, 240, and 300; high stream rate which comprises of last five stream rates i.e., 360, 420, 480, 540, and 600. For example, suppose the end-user application requires that results are produced with high stream data with higher latency irrespective of stream rate. In that case, the recommended window configuration is sliding with a large window size. Another example could be latency-sensitive scenarios where the recommended window configuration must be tumbling window with small window size. For the latency-sensitive cases and need a reasonable resulted data size, a sliding window with small window size configuration can compromise on these two contradicting requirements. On the other hand, in high stream data, the session window with large window size configuration can significantly gain a feasible result size while maintaining an acceptable latency. We built this knowledgebase based on our experiments, but it will gradually evolve as more, and more complicated scenarios occur during various query executions. This knowledgebase acts as the core component for the dynamic window configuration algorithm and recommendation of the optimal query window configuration (see Table 3).

Here we introduced our dynamic query stream processing algorithm-Dynamic Window Configuration (DWC) that integrates the discussed cost model based on stream data characteristics, application workload requirements and resource constraints. For simplicity, stream data characteristics including stream rate and application workload requirements are used. The DWC algorithm uses the historical statistics of the last executed windows to configure the window type and size of the fluctuated stream rates. Before we explain the DWC algorithm, we first introduce two data structures that are used to track the stream statistics and current window sizes. First, we use historical statistics denoted as Stream_Stat_Histo which contains Stream_Rate_Last to show the ingested stream rate and Window_Size_Last to record the last recommended window size for the last executed windows. The number of the last executed windows is configurable. A snapshot of historical statistics is described in JSON-like format as follows:

    {
    "Window"     : {
      "ProcessID" : "1" ,
      "JobName" :"AdpativeWindow" ,
      "Resources" : {
        "RAM" : 700 ,
        "CPU" : 4
      } ,
      "Requirements" : {
        "Latency" : "Low" ,
        "DataEnrichment" : "Low" ,
        "Accuracy" : "Low"
      } ,
      "Type" : "Tumble" ,
      "Size" : 1 ,
      "Duration" : 10 ,
      "StreamingProcessTime" : 125 ,
      "QueueingStreamingProcessTime" : 150 ,
      "StreamOut" : 600 ,
     "NumberofSensors" :10 ,
      "StreamIn" : {
       "sensor-value-1" :  600 ,
       "sensor-value-2" :  1200 ,
       "sensor-value-3" :  1000 ,
       "sensor-value-4" :  500 ,
       "sensor-value-5" :  800 ,
       "sensor-value-6" :  700 ,
       "sensor-value-7" :  400 ,
       "sensor-value-8" :  900 ,
       "sensor-value-9" :  1500 ,
       "sensor-value-10" : 300
       }
       }
       }

Second, we track the current window size using live statistics (denoted as Window_Size_Current) which indicates the configured window size for the current stream rate.

Algorithm 1 presents the core function that calculates the next window configuration including window type and size based on the recommended window configuration fetched from the knowledgebase. The input of the DWC algorithm is the Workload_Requirements_List which identified by the use case. First, the algorithm identifies the stream type using the *get_Stream_Type* function as shown in Line 6. The function of *get_Stream_Type* is used to map the numerical values of stream rate to the high-level representations of requirements within the built knowledgebase (e.g., stream rate = high). Secondly, the algorithm gets the window type and size from the built knowledgebase based on the workload requirements and stream rate type. Thirdly, for the window size, the algorithm decodes the high-level representations of window size into numerical values to be used for new window configuration. For the recommended large window size, the new window size will be increased. For the small window size, the new window size is identified according to the *StreamRateChangeRatio* stated on Line 14. The *StreamRateChangeRatio* is calculated based on the last stream rate and the current stream rate. Therefore, the new window size is rationally recommended based on the previous recommendation extracted from the knowledgebase, the status of both of current stream rate and the last executed window size(s).
**Algorithm 1:** Dynamic Window Configuration Algorithm (Simplified)
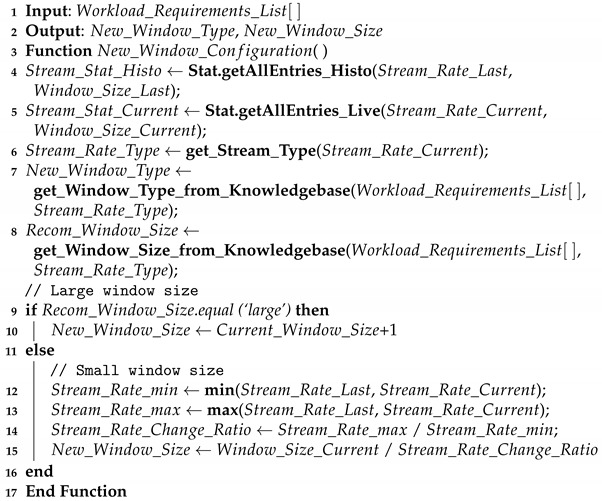


**Deployment phase.**

In the deployment phase, the new optimized stream query will be deployed using the selected window configuration based on the current stream job status, the stream rates, the application requirements, and resource constraints. In doing so, the new query will be deployed beside the current stream query (i.e., without killing the running query) and the current query still runs until the new one is warmed up.

## 6. Experimental Setup and Evaluation

The experiments have been conducted to perform data stream pipeline using Apache Kafka version 2.11–2.2.0, Apache Flink version 1.7, and MongoDB to consume data from data sources (i.e., sensor data generator), execute stream query and then store the reported data respectively. In the following subsections, the generation of forest fire dataset use case and the description of experiments and discussion are presented.

### 6.1. Forest Fire Based on Climate Change Dataset

We build our use case called forest fire detection to continuously monitor climate change, i.e., weather conditions in real-time (see Figure 1). As technical level, Apache Kafka is used to consume weather streaming data from the deployed sensors within a forest. The collected data (i.e., potential fire events) is then sent to the query engine running on the top stream processing platform (i.e., Apache Flink). The number of possible fire events is varying drastically during the day. In the normal weather conditions, the weather sensors will send lower data frequencies, and no action will be taken from the forest department authority side. For the forest fire situation, the weather sensors start to send data in higher frequencies which causes event-loss due to the delay in consuming and processing such massive streaming data. Therefore, the delay will raise late alarms to the forest department authority to send its firefighters and drones to the burning forest. Consequently, the dynamic query window selector occurs due to the delay issues caused by the static window configuration for stream query engines. The adaptive query window selector adapts the window size (i.e., increasing and/or decreasing window size according to the fluctuating stream rates during day hours). To the best of our knowledge, there is no open dataset of forest fire detection IoT based use case. The authors in [27] have developed a system with secure communication which has been configured for monitoring different variables of environments including temperature, humidity, CO, CO_2_ and wind speed. They have taken into account Rule of 30 that considers zones characterized by temperatures above 30 °C and humidity values below 30% as risk areas for forest fires. Authors in [25] have considered the probability of forest fire changing with temperature.

**Figure 14:**
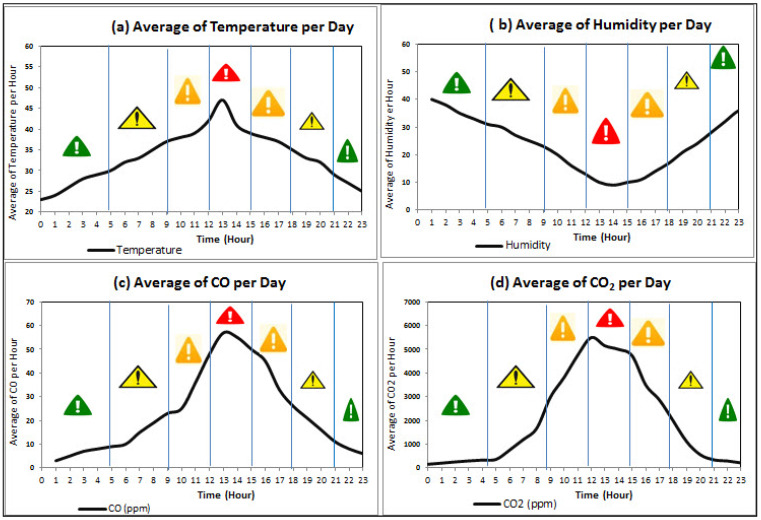
Average of sensor value per day generated using rule 30 and the corresponding alerts in uni-sensor environment: (**a**) temperature (**b**) humidity (**c**) CO (**d**) CO_2_.

**Figure 15:**
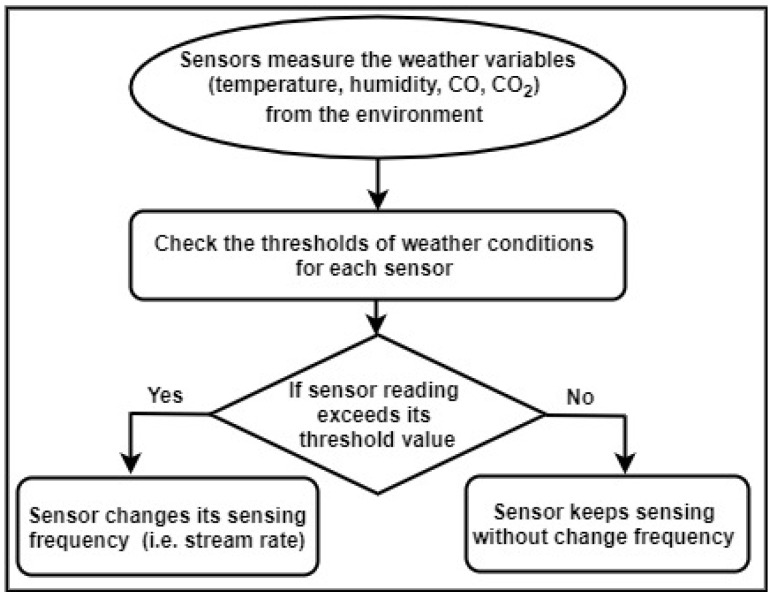
Forest fire use case dataset generator flowchart.

Based on the proposed work in [25,27], we prepare a dataset having five weather sensors including temperature, humidity, CO, CO_2_, and wind speed. The dataset is generated using Rule 30 to bring it close to reality, for example by showing temperature goes higher during the noon, late afternoon and lower after midnight [27,56] (https://en.wikipedia.org/wiki/Rule_30), (http://www.auburn.edu/academic/forestry_wildlife/fire/weather_elements.htm). However, the authors of [57] discussed the techniques and strategies for greening IoT by reducing the pollution hazards. Furthermore, we use the standard European Forest Fire Information System which describes the Forest Fire Danger Ratings into four categories; Green, Yellow, Orange, and Red (http://effis.jrc.ec.europa.eu) to define alert types thresholds for each sensor (see Figure 14). Our assumption for data generation is that each of the sensors remains in the thresholds defined for each alert type, e.g., when the temperature is less than 30, humidity is always greater than 30 and so on (see Table 4 and Figure 15).

In particular, we assume there are 100 nodes installed to produce weather sensor values (i.e., temperature, humidity, CO, and CO_2_) depending on the alert type threshold. For example, for green, yellow, orange and red, there are 2, 6, 12, and 60 values per minute sent from sensors respectively (see Table 5). The day is divided into different sections depending on the alert type, and in total there are 8 h of green, 7 h of yellow, 6 h of orange and 3 h of red. The rationale behind this assumption is that the midnight and early minoring hours are less likely to fire occurrences, i.e., green alerts. In contrast, the noon hours are the high probability for fire occurrence, i.e., red alerts. The sample of generated data for the weather variables values is provided in Table 6.

Substantially, high wind speed promotes supplying more oxygen from the surrounding environment. Therefore it increases the fire at a fast rate. Consequently, the wind speed, which probably has the most significant impact on a wildfire’s behavior, is the most unpredictable factor. Due to wind speed’s unpredictability during a day, we could not generate a wind speed stream using Rule 30 to be close to reality. We generate it within the speed range; between 1 and 20 km per hour [58].

### 6.2. Experimental Setup

We run six experiments for static window sizes including 1, 5, 10, 15, 30, and 60 s, using a tumbling window and one experiment for dynamic window size. Static window sizes experiments aim to show the static window’s impact and the stream rate changes on the reported alerts including the reported alerts, missed alerts and latencies. According to the latency setting, we ran a dynamic window size experiment to evaluate our proposed technique by adapting window size between 60 s and 1 s based on the input stream rate change. Then, we show the benefit of query window adaption in reducing the latency of reporting time while discussing the trade-offs regarding the alert losing due to the adaption process.

The conducted experiments execute a stream query that monitors alerts based on sensors’ values and then reports an event that is the alert type. In particular, the stream query joins two streams; weather stream including (temperature, humidity, CO, and CO_2_) and wind speed stream. Since not only the pattern of weather sensors thresholds is used to report the wildfires, the wind speed also is used to early notify the fires especially in case of the wind excess the high range, i.e., 20 km/h.

For brevity and simplicity, we run our experiments by setting the data generator to produce streaming data for 100 nodes within 24 min using the predefined stream rate of 24 h to make our experiments more tractable.

**Figure 16:**
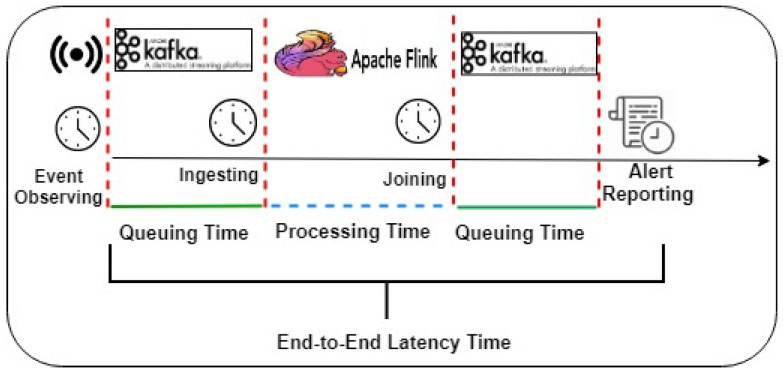
Timeline of stream-stream joins in the conducted experiments.

According to the listed experiments and the forest fire detection use case which concerns timely danger rating alerts, we measure these matrices; (1) output stream rate including the number of actual alerts, joined events/reported alerts, loss/missed alerts for each alerting category, (2) timeline latencies including queuing time, processing time, and end-to-end latency time (see Figure 16), (3) reduction of reporting time using dynamic window selector. For the output stream rate, the actual alerts measure the expected alerts resulted from join query based on the stream rate; the reported alerts measure the Flink resulted joined tuples, the loss/missed alerts measure the alerts which pass their time due to long waiting in Kafka queues. For the timeline latencies, the queuing time measures the consumed times by Kafka in two intervals; the first interval is when the event consumed from Kafka source (i.e., weather sensors) and the second interval is when the joined events/reported alerts are sent from Flink until reported (i.e., stored into MongoDB). The processing time measures when the event is ingested into Flink until the event was sent to Kafka again. The processing time also includes Flink join processing time which labels each event by the corresponding rating alert. The end-to-end latency time measures the time from the event is generated until the event/alert is reported, e.g., sent to the forest department authority.

### 6.3. Comparison of Different Static Window Sizes

**Figure 17:**
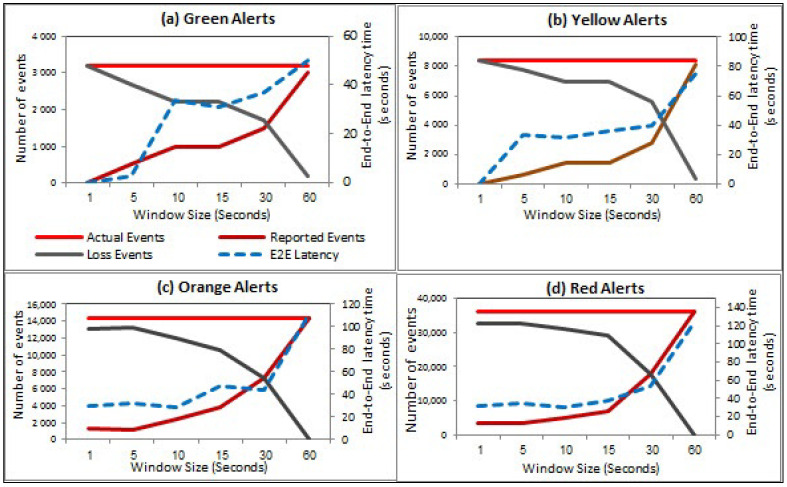
Comparison of static experiments for 100 nodes with respect to the number the reported events, the number of loss events and the end-to-end latency time.

The static window experiments that are conducted by fine-tuning the window sizes such as 1, 5, 10, 15, 30, and 60 s. For each window size, we first measure the number of actual alerts, joined events/reported alerts, loss events and the end-to-end latency time that can be incurred for each window size. Figure 17 showcase the linear increase of the reported alerts and decrease loss alerts for each type of alert concerning the actual alerts using different window sizes. It is noted that the number of reported alerts converges towards the number of the actual alert with window size increasing. In contrast, the number of loss alerts has the opposite behavior. We attribute this incremental behavior of the reported alerts to the increasing window size. More specifically, the large window size can hold many ingested events to be joined within a window.

Similarly, the decrease of loss alerts concerning the increasing window size is that the small window size fast joins the ingested events. Some queued events are lost by passing their timeout due to the long wait and couldn’t be joined with next windows. Thereby, the large window size can hold a larger number of input events that be joined and then reported. It can be seen that the average of end-to-end latency times gradually increases due to the increasing dormer size for each alert. For example, the green alert, which has the lowest stream rate, has the shortest latencies with respect to the other alerts for all window sizes. In contrast, the red alert, which has the largest stream rate, has the longest latencies with respect to the other alerts for all window sizes. The long latency of red alerts happens by increasing of stream rate over larger window size.

### 6.4. Comparison of Static and Dynamic Window Sizes Results

Based on the experimental results using static window sizes, we chose the static window size experiment of 60 s to compare with a dynamic window selection experiment which adapts the window size between 1 and 60 s. More specifically, this experiment has the lowest number of loss alerts concerning the other static window size experiments, making it the proper one to compare its performance with dynamic window selector performance. As the IoT-based forest fire monitoring use case is considered one of the latency-sensitive IoT-based application, the proposed dynamic window selector recommends the tumbling window type based on the built knowledgebase. Accordingly, the window size is initially configured using tumbling window and 60 s. The dynamic window selector slightly adapts the window size to maintain timely reported alerts for various stream rate while keeping the window type as tumbling because of its superiority for the latency reduction.

**Figure 18:**
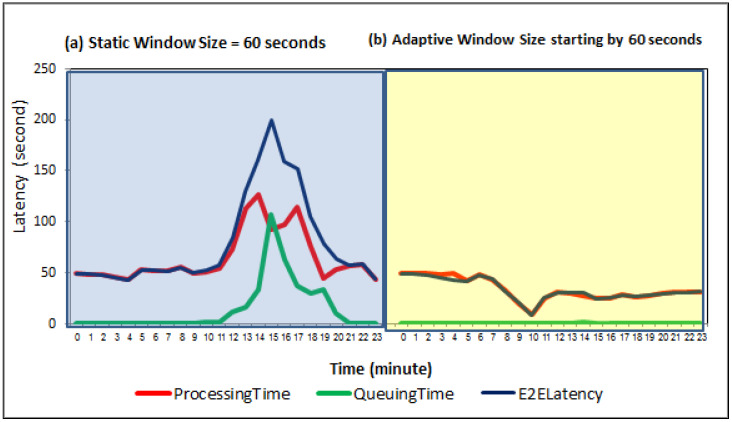
Comparison of static and adaptive window sizes for queuing time, processing time, end-to-end latency.

As can be seen, Figure 18a,b depict that the proposed window selector achieves lower latencies to the static window size. By introducing a streaming data pipeline, it is noticed that the latencies including queuing time, processing time and end-to-end latency time are significantly reduced compared to the static window size experiment by dynamically change window sizes for the sudden stream rates. Mainly, the dynamic window selector gradually decreases the window size based on the stream rate increments. The lower window size performs multiple short join intervals, leading to quickly consuming data from Kafka and decreasing the queuing time (see Figure 18b).

**Figure 19:**
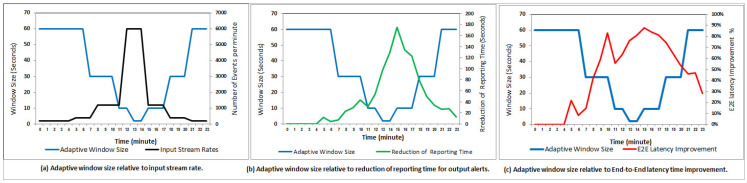
Dynamic experiment number 7. (**a**) Dynamic window size relative to input stream rate (**b**) dynamic window size relative to reduction of reporting time for output alerts (**c**) dynamic window size relative to End-to-End latency time improvement (E2ELatency).

Considering the reality of the desired contribution, Figure 19a shows that our dynamic window selector can adapt the window size according to time-varying input stream data rates based on 24-h monitoring of weather conditions in the forestry areas. For afternoon hours (i.e., 12, 13, and 14 o’clock) which are the most latency-sensitive for fire probabilities, our dynamic window selector can achieve the highest reporting delay reduction relative to the rest hours per day (see Figure 19b). Furthermore, Figure 19c shows the significant end-to-end latency time, E2E, improvement relative to window size which leads to faster alerting the forest department authority to protect the forests from fire spreading and huge damages.

**Figure 20:**
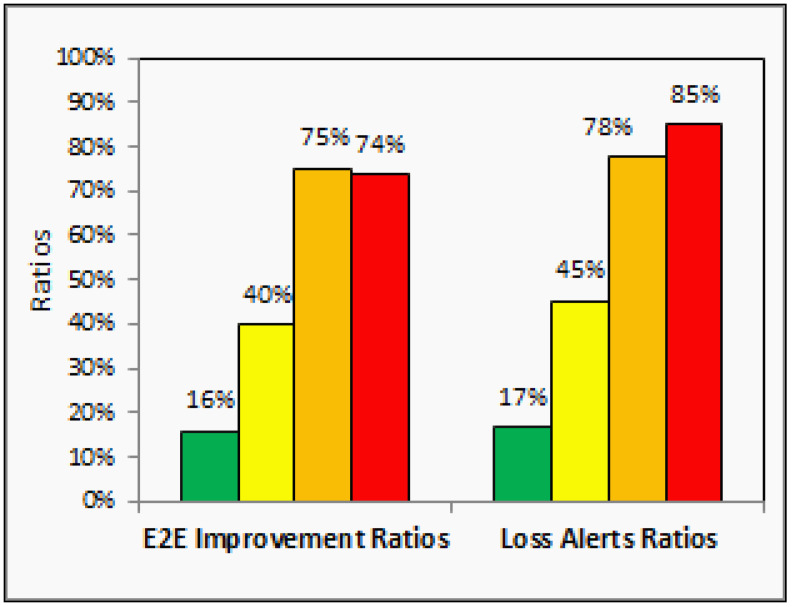
Summary of end-to-end latency time improvement (E2E) and loss alerts ratios for each alert type for adaptive window size experiment with respect to the static window experiment.

In Figure 20, we analytically and experimentally summarize the performance gained from window size adaptation, showing their superiority over the static window size configuration for each danger rating alert. However, the results show the trade-off between the end-to-end latency time improvements and the loss alerts ratios. For example, the red alerts have the highest number of loss alerts concerning another type of alerts which is on average is 85%. Still, its reporting time (i.e., end-to-end latency time improvement) is reduced by 74%. In particular, the proposed dynamic window selector has deployed a query with a smaller window size to perform fast joins using a smaller number of ingested events to trigger the early red alerts. The early notification using a smaller number of ingested events is better than deploying a query with a larger window size to perform late joins using delayed events having similar sensors values. As the selected use case, IoT-based forest fire detection, considered one of the latency-sensitive applications, we believe that losing 85% of red alerts is still reasonable to provide a timely warning about fires by using 15% of the expected alerts while reducing notification time to 74%. Finally, our experimental results over the generated forest fire dataset confirm that the dynamic window selector significantly reduces the end-to-end latency time for the reported fire alerts by sending fast danger alerts even though there are many missed alerts during the adaptation process.

## 7. Discussion

To bring the static window size experimental investigation to reality, the weather variables including temperature, humidity, CO, CO_2_ and wind, are considered relying on the new technology of IoT, which can achieve 24-h monitoring of weather conditions. From this experimental investigation, we learn that the large window size is suitable for the low stream rate, which reports the green alerts in normal weather conditions. On the other hand, the small window size which reports fast alerts is good for the high stream rate, which leads to critical danger ratings (i.e., red alerts). In terms of forest fire detection, it is a widely accepted view that relies on how difficult to detect forest fires accurately using static streaming window configurations. Consequently, the window size needs to linearly adapt according to the increasing stream rate to keep maintaining the critical alerts even with a reasonable number of loss alerts that indicate forest fires. In particular, we need to deploy a suitable dynamic streaming window configuration to maintain the weather data changes’ stream rates to avoid the potential fires in the broad forestry areas, especially in the summer season.

In summer times which are usually called fire seasons, more than 80% forest fire occurred either in the spring or in the autumn with a slight increasing distribution due to the drier weather conditions [25]. According to this work, the weather variables, including temperature, humidity, CO, CO_2_, and wind, are considered relying on IoT’s new technology, which can achieve 24-h monitoring of weather conditions in forestry areas. The joined streaming results, which are the alerts, can illustrate the relationships between the weather alert thresholds and the wind speed. Therefore, based on the findings using our proposed dynamic window-based selection system, the reported alerts are very time-sensitive in case of high wind speed. In particular, in the autumn with the drier weather conditions, the orange alerts should be considered red alerts in high wind speed, which strongly warns of danger fires igniting. Consequently, our proposed dynamic window selection system can inform the forest fire authorities about the forest fires that rely on different weather conditions even not fire seasons.

### 7.1. Challenges

In this paper, we present a novel dynamic query window-based processing system to recommend the optimal window size and type based on the dynamic context of IoFT applications. Regarding improving forestry 4.0 environmental sustainability, real-time network connectivity of IoFT devices and decision making will enhance forest resources to be continuously managed and monitored. Management of forestry 4.0 will be easy due to reducing resource damages and wastes, while harvesting will be coordinate to minimize gas emissions. Autonomous IoFT devices, vehicles and robots will improve safety and also working environment. There are more complex queries with complex operations, e.g.; multiple streams join since such cases/scenarios are non-trivial to solve and may consider for future work. Furthermore, The delay of reporting time in case of missed and delayed events could be reduced by identifying the optimal amount of resources to satisfy the required processing delay under specific stream rate change [59]. In integration with other adaptive streaming techniques, other adaptive techniques such as adaptive load shedding for windowed stream joins [13] and dynamic batch sizing [17] can be integrated with our proposed dynamic windowing based on stream rate change for industrial applications. For stream processing platforms, the streaming data processing results should be on-the-fly to support alerting applications that process new data at the speed with which it is generated. Event streams are potentially unbounded, infinite sequences of records and unpredictable that represent events or changes in real-time. Consequently, the in-memory stream processing platforms must be both fast and scalable to handle billions of tuples every second. Memory consumption is one of the scalability challenging aspects that affect stream processing performance, especially for alerting applications, i.e., publishing notifications to subscribers. The existing research of adaptive windowing lacks solutions that consider memory consumption with the variable stream rates generated from the event sources (e.g., applications and sensors) and the critical destinations such as an alerting system. Regarding our proposed system, changing the window size can reduce the trade-offs with memory consumption. For example, assigning small window sizes for high stream rates helps avoid data drift problems (i.e., memory crash) and meet analytics requirements such as latency constraints. For further investigation, we plan to address the bottleneck and performance problems briefly due to memory consumption during streaming data processing [60].

### 7.2. Opportunities

We believe that the proposed system has taken a number of steps in this direction. Furthermore, the future work will take us much closer to comprehensive solutions for variable size windowing over IoT-based stream rate change. Moreover, the proposed system able to manage the sudden large change in stream rate to establish a set of early warning mechanisms for the quick response for critical-latency applications. Gathering data and producing accurate alert of fire detection in Forestry 4.0 requires to distribute a massive number of smart devices and efficient processing techniques. Real-time fire detection and management alert is a critical issue for improving Forestry 4.0 application implementation. Therefore, this issue should be given high consideration to improve treating with a real situation and supporting needed real remedies to situations in real-time. Furthermore, distributed several kinds of smart devices could be used to monitor the forest fire. Heterogeneous smart devices gather different data. Applying a multi-model for gathered data is also a critical issue for forest fire detection and management.

Transmission gathered data for long-distance is a critical issue for improving Forestry 4.0 monitoring. Therefore, processing gathered data locally should be taken into consideration for improving forest situation in real time. Another solution, drone technology may be taken into consideration as edge station for gathering data from closed distance to smart devices efficiently and line of sight [61,62]. Furthermore, drones can process data nearest to smart devices and take action based on an algorithm implemented by using deep learning and store in the drone. in this case, drones can produce alerts of fire detection in forests. On the other hand, there is an opportunity to use edge technology to improve the economy and manage wood production (i.e., drone edge intelligence for gathering data from IoFT devices).

## 8. Conclusions

Industry 4.0 is gaining advantages from the implementation in different domains. Therefore, the transformation of Industry 4.0 towards Forestry 4.0 in the forest domain appears inevitable. The advantages of this transformation will significantly improve the social, environmental and economical. Therefore, Forestry 4.0 is a promising sector of Industry 4.0 to make a real sustainable revolution. We applied a dynamic window configuration recommendation techniques to handle variable stream rate and build an optimal query execution plan. This approach shows a significant enhancement on stream query optimization. Furthermore, it presents additional modules, such as input stream monitoring and creates an open knowledge base. This addition plays a vital role in evaluating the optimal window configuration. Regarding forest fire detection and management by using IoFT, we describe how IoFT generates streaming datasets for a rapidly changing ecological environment. The forest fire dataset is evaluated using static and dynamic window sizes configurations. The proposed dynamic window size selector shows its superiority to send timely alarming alerts by adapting window size based on the sudden stream rate changes.

Forestry 4.0 has a bright and exciting future. In the future, the extent of this work will be in the augment our approach within top distributed stream processing platforms. Build an open knowledgebase/knowledge graph storing statistics of IoT devices’ performance. Their stream rates can be utilized by multiple platforms and applications to build robust and adaptive IoT applications. Memory consumption and connectivity among smart IoFT devices are the limitation of our proposed approach. It will be our future direction to improve sustainability impacts and manage fire detection before it occurs. On the other hand, improving dynamic selectors to predict future environmental risks will help identify and detect risk before it occurs by collecting large dataset from high input stream rates and then applying powerful predictive analytics techniques such as deep machine learning. Moreover, the combination of deep learning and blockchain technology can be used in order to improve processing, resource utilization and connectivity among smart IoFT devices. Blockchain is a decentralized technology for smart devices and makes decisions locally instead of sending data to the center for analysis. While, deep learning can be implemented in smart contracts to process data locally and make decision making based on historical storage data in blockchain to produce fire alert immediately.

## Figures and Tables

**Table 1:** Summary of the related research work.

Ref. (Year)	Focused	Advantages	Industry 4.0	IoT	Forestry 4.0	Fire Detection	Impacts			
							Social	Ecological	Economical	Environmental
[31] (2020)	IoT for improving logistical processes of global wood processing	- Reducing operating costs - Increasing added value - Collecting real-time data	✓	✓					✓	✓
[8] (2019)	Forest operations and proposed sustainability impact assessment	- Improving efficiency - Improving safety - Environmental protection					✓		✓	✓
[44] (2018)	Fuzzy logic for forest fire detection, prevention based WSN	- Enhancing emergency response time - Estimating the forest fire risks		✓		✓				✓
[30] (2019)	Internet of things and digital twins for improving forestry 4.0	- Improving connectivity of forestry 4.0 and software center	✓	✓	✓				✓	✓
[38] (2019)	Digitization in wood supply	- Improving the impacts including social, ecological, economic, and environmental	✓				✓	✓	✓	✓
[45] (2008)	Sustainable forest management meeting ecological, economic and social needs	- Trade-off of sustainable components					✓	✓	✓	
[46] (2016)	Analyzing the effects of sustainable forest management	- Resource management					✓		✓	✓
[47] (2018)	Maximum sustainable woody biomass harvest potential	- Reducing carbon-dioxide forest sequestration from the atmosphere							✓	✓
[46] (2016)	Analyzing the effects of sustainable forest management	- Resource management					✓		✓	✓
[47] (2018)	Maximum sustainable woody biomass harvest potential	- Reducing carbon-dioxide forest sequestration from the atmosphere							✓	✓
[40] (2020)	Framework industry 4.0 toward forestry 4.0 of the forest supply chain	Significant economically, socially, and environmentally of industry transformation framework for the forest supply chain toward Industry 4.0	✓	✓	✓		✓	✓	✓	✓

**Table 2:** Comparison of industrial research in forest fire detection domain.

Ref. (Year)	Ref	Highlighted	Technologies			Forestry 4.0	Industry 4.0	Applications		
			IoT	WSN	IoFT			General	Monitoring	Fire Detection
[30] (2019)	Industry 4.0 beyond the Factory: An Application to Forestry	Connecting the forestry to central software with the help of digital twins	✓				✓	✓		
[44] (2018)	Forest Fire Prevention, Detection, and Fighting Based on Fuzzy Logic and Wireless Sensor Networks	Forest Fire Prevention, Detection, and Fighting	✓	✓		✓		✓	✓	✓
[25] (2018)	A fuzzy inference and big data analysis algorithm for the prediction of forest fire based on rechargeable wireless sensor networks	A fuzzy inference for the prediction of forest fire based on wireless sensor networks		✓				✓	✓	✓
[53] (2017)	The Internet of Things– wireless sensor networks and their application to forestry	IoT for forestry applications	✓	✓					✓	
[54] (2020)	Smart forests and data practices: From the Internet of Trees to planetary governance	Internet of tree connected with government	✓						✓	
[39] (2020)	Internet of Things for Sustainable Forestry	IoT and communication applications for sustainable forestry systems	✓		✓				✓	
[6] (2014)	IoT Forest Environmental Factors Collection Platform Based on ZIGBEE	Monitor the forest environmental factors using the ZIGBEE protocol with the new IoT technology	✓	✓	✓				✓	
[27] (2017)	Management of Forest Fires Using IoT Devices	Using of IoT technologies to prevent forest fires	✓		✓	✓		✓	✓	
[26] (2018)	IoT-based intelligent modeling of smart home environment for fire prevention and safety	Global System for Mobile Communications (GSM) to avoid false alarms by simulating a fir in a smart home prevention and safety	✓		✓			✓	✓	
[40] (2020)	Framework industry 4.0 toward Forestry 4.0 of the forest supply chain	Forest supply chain toward Industry 4.0 in terms of economic, society and environment	✓			✓	✓	✓		

**Table 3:** Recommended stream query window configurations for some use cases requirements i.e., latency and data enrichment.

Usecase. No	Requirements Description	Workload Requirements		Stream Rate	Recommended Window Type	Recommended Window Size
		Latency	Data Enrichment			
1	data enrichment requirement has the higher priority	-	High	Low	Sliding	Large
2	data enrichment requirement has the higher priority	-	High	High	Sliding	Large
3	Latency requirement has the higher priority	Low	-	Low	Tumbling	Small
4	Latency requirement has the higher priority	Low	-	High	Tumbling	Small
5	Latency requirement has the higher priority and reasonable resulted data size is needed	Low	Medium	Low	Sliding	Small
6	Latency requirement has the higher priority and reasonable resulted data size is needed	Low	Medium	High	Sliding	Small
7	Latency requirement and data enrichment have the same priority	Medium	Medium	Low	Sliding	Small
8	Latency requirement and data enrichment have the same priority	Medium	Medium	High	Session	Large

**Table 4:** Weather variable alert thresholds.

Weather Variables	Green	Yellow	Orange	Red
Temperature	<30 ${}^{\circ }$C	≥30	≥37 ${}^{\circ }$C	≥40 ${}^{\circ }$C
Humidity	>30%	≤30%	≤20%	≤10%
CO (ppm)	<10	≥10	≥25	≥50
CO_2_ (ppm)	<350	≥350	≥2000	≥5000

**Table 5:** The input and output stream rates per minute, the corresponding fire alert during day and night hours for one node.

Fire Alert Type	Hours	Input Stream Rate	Output Stream Rate
Green	[0–4, 21–23]	2	4
Yellow	[5–8, 18–20]	6	12
Orange	[9–11, 15–17]	12	24
Red	[12–14]	60	120

**Table 6:** Sample of weather variables values per day generated for one node using rule 30.

Hour	Avg. Temperature	Avg. Humidity%	Avg. CO (ppm)	Avg. CO_2_ (ppm)
0	23	40	3	152
1	24	38	5	201
2	26	35	7	250
3	28	33	8	295
4	29	31	9	330
5	30	30	10	355
6	32	27	15	750
7	33	25	19	1191
8	35	23	23	1678
9	37	20	25	2986
10	38	16	36	3811
11	39	13	48	4750
12	42	10	57	5500
13	47	9	55	5150
14	41	10	50	5002
15	39	11	45	4730
16	38	14	33	3500
17	37	17	26	2870
18	35	21	21	1999
19	33	24	16	1112
20	32	28	11	560
21	29	32	8	340
22	27	36	6	290
23	25	37	4	210

## Data Availability

No Data available online. For further query email to corresponding author (radhya.sahal@nuigalway.ie, radhya.sahal.dsi@gmail.com).
